# Identification of Differentially Expressed Genes in COVID-19 and Integrated Bioinformatics Analysis of Signaling Pathways

**DOI:** 10.1155/2021/2728757

**Published:** 2021-12-24

**Authors:** Linjie Fang, Tingyu Tang, Mengqi Hu

**Affiliations:** ^1^Rehabilitation Assessment and Treatment Center, Zhejiang Rehabilitation Medical Center, No. 2828 Binsheng Road, Binjiang District, Hangzhou 310000, Zhejiang, China; ^2^Department of Respiratory, Zhejiang Hospital, No. 12, Lingyin Road, Hangzhou 310000, Zhejiang, China; ^3^Respiratory Examination Center, Department of Respiratory, Zhejiang Hospital, No. 12, Lingyin Road, Hangzhou 310000, Zhejiang, China

## Abstract

Coronavirus disease 2019 (COVID-19) is acutely infectious pneumonia. Currently, the specific causes and treatment targets of COVID-19 are still unclear. Herein, comprehensive bioinformatics methods were employed to analyze the hub genes in COVID-19 and tried to reveal its potential mechanisms. First of all, 34 groups of COVID-19 lung tissues and 17 other diseases' lung tissues were selected from the GSE151764 gene expression profile for research. According to the analysis of the DEGs (differentially expressed genes) in the samples using the limma software package, 84 upregulated DEGs and 46 downregulated DEGs were obtained. Later, by the Database for Annotation, Visualization, and Integrated Discovery (DAVID), they were enriched in the Gene Ontology (GO) terms and Kyoto Encyclopedia of Genes and Genomes (KEGG) pathways. It was found that the upregulated DEGs were enriched in the type I interferon signaling pathway, AGE-RAGE signaling pathway in diabetic complications, coronavirus disease, etc. Downregulated DEGs were in cellular response to cytokine stimulus, IL-17 signaling pathway, FoxO signaling pathway, etc. Then, based on GSEA, the enrichment of the gene set in the sample was analyzed in the GO terms, and the gene set was enriched in the positive regulation of myeloid leukocyte cytokine production involved in immune response, programmed necrotic cell death, translesion synthesis, necroptotic process, and condensed nuclear chromosome. Finally, with the help of STRING tools, the PPI (protein-protein interaction) network diagrams of DEGs were constructed. With degree ≥13 as the cutoff degree, 3 upregulated hub genes (ISG15, FN1, and HLA-G) and 4 downregulated hub genes (FOXP3, CXCR4, MMP9, and CD69) were screened out for high degree. All these findings will help us to understand the potential molecular mechanisms of COVID-19, which is also of great significance for its diagnosis and prevention.

## 1. Introduction

Coronavirus disease 2019 (COVID-19) represents pneumonia created by a new type of coronavirus infection in 2019, which is a type of acutely infectious pneumonia [1]. The disease can be spread through respiratory tract saliva and contact and is highly infectious [2]. According to real-time statistics, about 6:30 on May 13, 2021, Beijing time, a total of 161,032,168 cases of new coronary pneumonia had been confirmed globally, and the death toll was as high as 3,343,853 [3]. The main clinical manifestations are fever, dry cough, fatigue, and other symptoms [4]. The diagnostic methods currently approved for popularization include nucleic acid detection reagents and antibody detection reagents. Chest X-rays, chest CT, blood routine, blood gas analysis, myoglobin, and other medical tests can also be used as auxiliary testing methods [5]. At present, most mild and ordinary patients can be treated symptomatically with antiviral and antibacterial drugs and immunotherapy, but the prognosis of severe patients is not optimistic. Fortunately, with the continuous deepening of COVID-19 research, many countries have developed a variety of vaccines to prevent COVID-19 after a large number of clinical trials [6]. However, due to the mutation of COVID-19, it is still very difficult to determine treatment plans. So, it is crucial at present to delve into the potential molecular mechanisms of COVID-19 and explore more effective prevention measures, diagnosis techniques, and treatments.

DNA microarrays are made by printing the oligonucleotide probes onto special glass slides. Tens of thousands of nucleic acid probes are contained on an area of a few square centimeters. They can provide a lot of gene sequence-related information [7]. As a tool for genomics and genetics research, DNA microarrays are widely used to detect gene expression levels, identify gene sequences, and discover new genes and other related methods. Matsuzaki et al. used DNA microarray analysis to study the pathophysiology of endometriosis [8]. Brandt et al. detected mRNA expression levels of genes in pancreatic duct adenocarcinoma based on DNA microarray technology, which provides a direction for improving the poor prognosis and discovering new molecular markers and therapeutic targets [9]. Guo mentioned that the completion of human genome sequencing and the advancement of DNA microarray technology have accelerated the progress of genetic analysis on a genome-wide scale [10]. This method promotes a better understanding of the underlying mechanism of tumorigenesis and provides more effective therapeutic interventions. With the prevalence of microarray technology, a large number of data have been accessible, and the integration can conduct more in-depth research on molecular mechanisms.

The purpose of this research is to find potential therapeutic targets related to COVID-19. First, we selected 51 samples from the GSE151764 gene expression profile as the research object and screened the DEGs using the limma package. Afterwards, the Database for Annotation, Visualization, and Integrated Discovery (DAVID) was used for analyzing the enrichment of DEGs in Gene Ontology (GO) and Kyoto Encyclopedia of Genes and Genomes (KEGG) pathways. Then, with the help of gene set enrichment analysis (GSEA), the enrichment of the gene set in the sample was analyzed. Finally, the protein-protein interaction (PPI) network of DEGs was designed for the hub genes' identification. The results of the above studies will offer new clues for the COVID-19 treatment and reveal the relationship between COVID-19 and the lung.

## 2. Materials and Methods

### 2.1. Microarray Data

A DNA microarray includes three parts: oligonucleotide chip, cDNA chip, and genome chip. It is a new technology for analyzing genome and gene expression profiles. It is also used to detect gene expression levels, identify gene sequences, discover new genes, and perform genetic mapping. The Gene Expression Omnibus (GEO, https://www.ncbi.nlm.nih.gov/geo/) database belongs to the National Center for Biotechnology Information (NCBI) [11] and is presently the largest and most comprehensive gene expression database, which mainly includes microarray and transcriptome sequencing data. We acquired GSE151764 in the GEO database. The expression profile contained 56 sample data. We selected 34 groups of COVID-19 lung tissues as the experimental group and 17 groups of lung tissues from other diseases as the control group for research.

### 2.2. Identification and Screening of DEGs

In order to analyze the differentially expressed genes (DEGs) between COVID-19 lung tissues and other lung tissues, we used R language software to obtain the DEGs in the sample data with the aid of the limma software package. *P* < 0.05 and fold change (FC) >2 were used as the screening criteria for upregulated DEGs and *P* < 0.05 and FC <0.5 for downregulated DEGs.

### 2.3. Enrichment Analysis by GO and KEGG Pathways

DAVID (http://david.abcc.ncifcrf.gov/) is a biological information database that integrates biological data and analysis tools, which can be used to identify GO entries, gene ID conversion, gene function enrichment analysis, etc. GO is a database established by the Gene Ontology Federation to comprehensively describe the attribution of genes and gene products in organisms, molecular function (MF), biological process (BP), and cellular components (CC) included. KEGG is a comprehensive database that integrates genomic, chemical, and systematic function information. One of the databases is called KEGG pathway, which is specifically used to store information about gene pathways in different species. In this study, based on the above tools, we analyzed the enrichment of DEGs in GO and KEGG, and *P* < 0.05 was considered statistically significant.

### 2.4. GSEA

GSEA (http://www.gsea-msigdb.org/gsea/index.jsp) is based on the use of a predefined gene set, sorting according to the differential expression of genes in the sample, and checking whether the predefined gene set is enriched at the top or bottom of the sorting table. In this study, we tested the GO term of the gene set enrichment in the samples based on GSEA.

### 2.5. Construction of the PPI Network and Identification of Hub Genes

The Search Tool for the Retrieval of Interacting Genes (STRING, http://string-db.org) database is a study of the interaction network between proteins. Cytoscape is software that concentrates on open-source network visualization and analysis. A simple network graph includes nodes and edges; each node represents a different protein gene. We constructed the PPI network of DEGs by the STRING database, visualized it with Cytoscape, and screened out the hub genes based on the degree of the gene.

## 3. Results

### 3.1. The Microarray Data Information and DEGs' Identification

**Figure 1:**
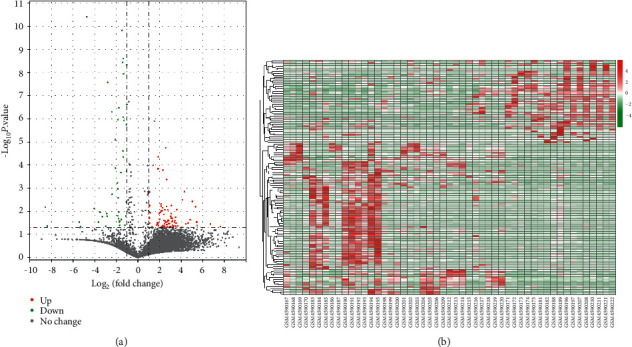
Volcano map and heat map of DEGs. (a) Volcano map. Green stands for downregulated DEGs, red stands for upregulated DEGs, and gray stands for no significant change genes. (b) Heat map. The expression levels of DEGs in 51 samples.

Limma package in R was performed on the GSE151764 dataset, and 130 DEGs between 34 COVID-19-caused death lung tissue samples and 17 other disease-caused death lung tissue samples were comprehensively screened out, including 84 upregulated DEGs (*P* < 0.05, FC > 2) and 46 downregulated DEGs (*P* < 0.05, FC < 0.5). In Figure 1(a) volcano map, the green part represented downregulated DEGs, the red part represented upregulated DEGs, and the gray part represented no significantly changed genes.  Figure 1(b) shows the distribution of DEGs in the 51 samples. The top 10 most significant DEGs are listed in Supplementary Table 1.

### 3.2. GO Term Enrichment Results of DEGs

**Figure 2:**
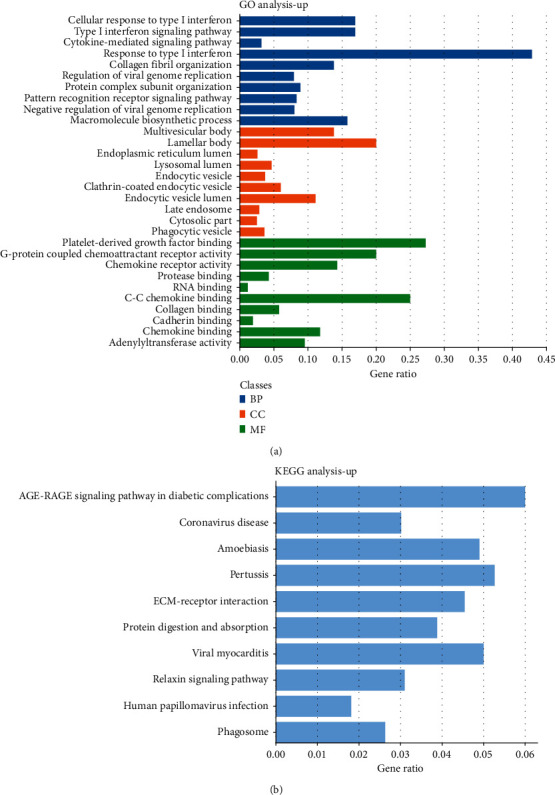
Upregulated DEGs' analysis. (a) GO analysis. Blue represents BP, orange represents CC, and green represents MF. (b) KEGG pathway enrichment analysis.

**Figure 3:**
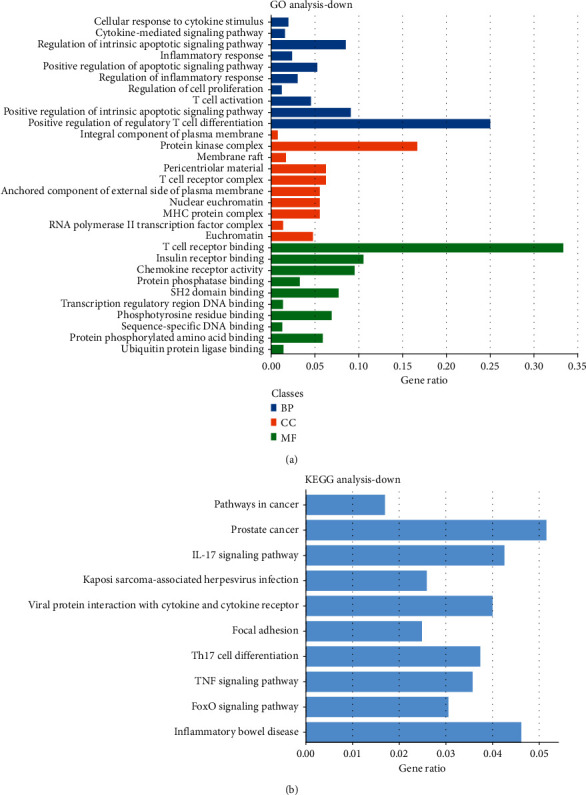
Downregulated DEGs' analysis. (a) GO analysis. Blue represents BP, orange represents CC, and green represents MF. (b) KEGG pathway enrichment analysis.

Based on the DAVID online analysis tool, the DEGs in 51 samples were bio-annotated. Under the condition of *P* < 0.05, the GO term enrichment results of DEGs were obtained.  Figure 2(a) shows the enrichment results of upregulated DEGs in the GO term. Upregulated DEGs were enriched in cellular response to type I interferon, type I interferon signaling pathway, cytokine-mediated signaling pathway, response to type I interferon in BP, multivesicular body, lamellar body, endoplasmic reticulum lumen, lysosomal lumen, endocytic vesicle, etc., in CC and platelet-derived growth factor binding, G-protein-coupled chemoattractant receptor activity, chemokine receptor activity, protease binding, RNA binding, etc., in MF.  Figure 3(a) shows the GO term results of downregulation of DEG enrichment. In BP, downregulated DEGs were enriched in cellular response to cytokine stimulus, cytokine-mediated signaling pathway, regulation of the intrinsic apoptotic signaling pathway, inflammatory response, positive regulation of the apoptotic signaling pathway, etc., in BP, protein kinase complex, membrane raft, pericentriolar material, T-cell receptor complex, anchored component of the external side of the plasma membrane, etc., in CC, and T-cell receptor binding, insulin receptor binding, chemokine receptor activity, protein phosphatase binding, SH2 domain binding, etc., in MF.

### 3.3. Analysis of the KEGG Pathway Related to DEGs

In the KEGG pathway enrichment analysis, upregulated DEGs were significantly enriched in the AGE-RAGE signaling pathway in diabetic complications, coronavirus disease, amoebiasis, pertussis, ECM-receptor interaction, protein digestion and absorption, viral myocarditis, relaxin signaling pathway, human papillomavirus infection, and phagosome (Figure 2(b)). Downregulated DEGs were in pathways in cancer, prostate cancer, IL-17 signaling pathway, Kaposi sarcoma-associated herpesvirus infection, viral protein interaction with cytokine and cytokine receptor, focal adhesion, Th17 cell differentiation, TNF signaling pathway, FoxO signaling pathway, and inflammatory bowel disease (Figure 3(b)).

### 3.4. GSEA

**Figure 4:**
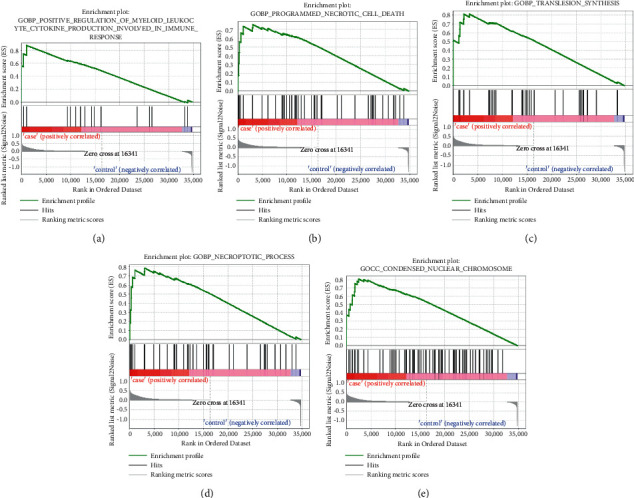
GSEA showed GO terms related to COVID-19-caused death lung tissue samples. (a) Positive regulation of myeloid leukocyte cytokine production in the immune response. (b) Programmed necrotic cell death. (c) Translesion synthesis. (d) Necroptotic process. (e) Condensed nuclear chromosome.

Figure 4 shows the GO terms related to COVID-19 through GSEA. We obtained the COVID-19-caused death gene set which was enriched in BP in positive regulation of myeloid leukocyte cytokine production involved in immune response (Figure 4(a)), programmed necrotic cell death (Figure 4(b)), translesion synthesis (Figure 4(c)), and necroptotic process (Figure 4(d)) and enriched in CC in the condensed nuclear chromosome (Figure 4(e)).

### 3.5. PPI Network of DEGs and Identification of Hub Genes

**Figure 5:**
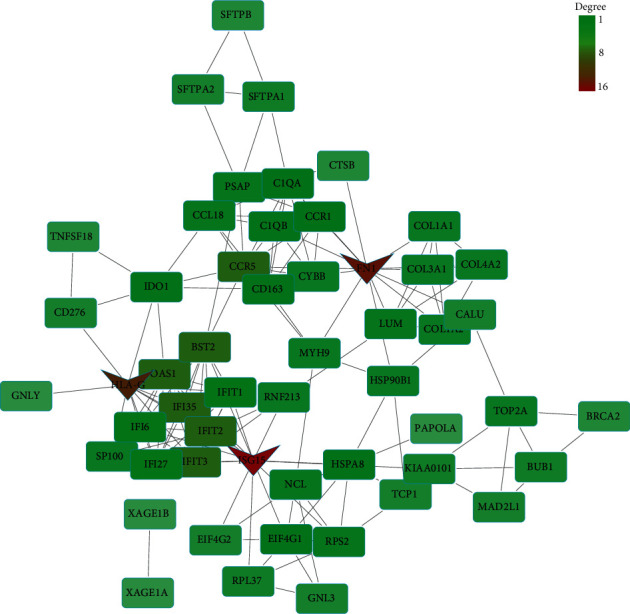
PPI network of upregulated DEGs. The PPI network consists of 53 nodes and 158 edges. Nodes represent genes, and edges represent interactions between genes. The darker-colored nodes represent the hub genes, which are ISG15, FN1, and HLA-G.

**Figure 6:**
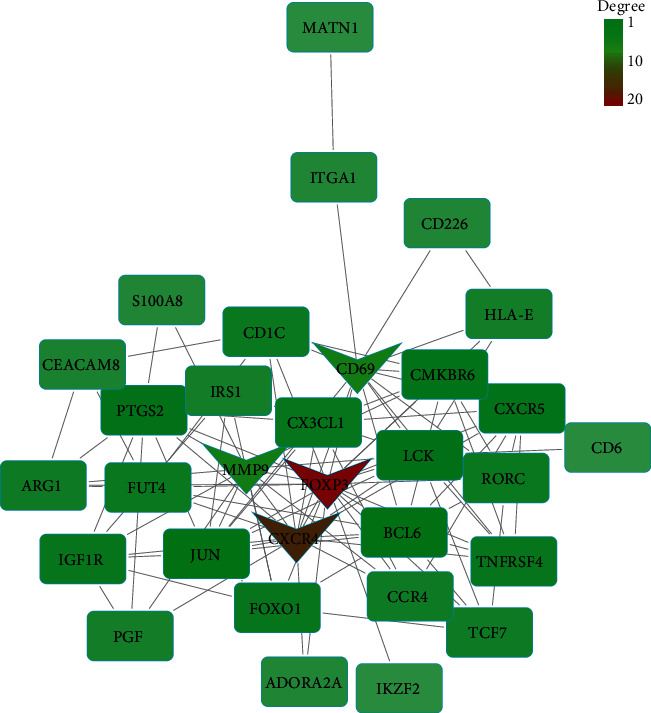
PPI network of downregulated DEGs. The PPI network consists of 31 nodes and 103 edges. Nodes represent protein genes, and edges represent interactions between genes. The darker-colored nodes represent the hub genes, which are FOXP3, CXCR4, MMP9, and CD69.

Based on the STRING database, we constructed a PPI network for upregulating DEGs and downregulating DEGs.  Figure 5 is a PPI network of upregulated DEGs. The network comprises 53 nodes and 158 edges. Each node represented a different protein gene, and the edges represented the connectivity between genes. With degree ≥13 as the cutoff criterion, we screened out 3 upregulated hub genes, namely, ISG15 (degree = 16), FN1 (degree = 14), and HLA-G (degree = 13). The PPI network of downregulated DEGs contained 31 nodes and 103 edges (Figure 6). With degree ≥13 as the cutoff degree, 4 hub genes were screened out, namely, FOXP3 (degree = 20), CXCR4 (degree = 16), MMP9 (degree = 13), and CD69 (degree = 13).

## 4. Discussion

COVID-19 is the main medical problem threatening human beings today. It spreads very fast, and the virus has also mutated in some countries, resulting in an increasing number of deaths. According to statistics from the World Health Organization, until May 2021, five countries, the United States, Brazil, India, the United Kingdom, and Mexico, had the highest number of deaths [12]. Due to the long incubation period of COVID-19, it is often accompanied by mild dry cough, fatigue, shortness of breath, fever, and diarrhea in the early stage, which cannot attract people's attention and cause a large amount of transmission. In addition, COVID-19 can cause brain damage, stroke, encephalopathy, encephalitis, peripheral neuropathy, and other complications. Critically ill patients will develop a diffuse alveolar injury, respiratory failure, acute respiratory distress syndrome, etc. The treatment process is very difficult [13]. Therefore, studying the important molecular mechanisms of the occurrence and development of COVID-19 is of great significance not only to individuals but also to all mankind.

Microarray and high-throughput sequencing technologies have been applied to the research of COVID-19, providing many new ideas for clinical defense and treatment [14]. So far, there is still no cure for COVID-19, and the prognosis is still not optimistic. Therefore, in this study, we analyzed the 34 groups of COVID-19 lung tissues as the experimental group and the other 17 groups of lung tissues in the GSE151764 gene expression profile based on microarray analysis and searched for COVID-19-related new therapeutic targets. First of all, we screened 84 upregulated and 46 downregulated DEGs from these samples. Afterwards, the enrichment of these DEGs in the GO terms and KEGG pathways was analyzed through the DAVID database. As a result, the upregulated DEGs were enriched in cellular response to type I interferon, type I interferon signaling pathway, coronavirus disease, etc., and downregulated DEGs in cellular response to cytokine stimulus, inflammatory response, IL-17 signaling pathway, FoxO signaling pathway, etc. Park described in the study that type I interferon activates the adaptive immune response to the virus which could be used as a potential application of new coronary pneumonia treatment strategies [15]. Hawkes et al. found that the inflammatory response produced by IL-17 T cells produced in the mouse autoimmune model promotes the development of skin lesions in the inflammatory cell population [16]. Besides, the IL-17 signaling pathway is involved in the pathogenesis of plaque psoriasis and occupies an important role. Farhan et al. studied the progress of FoxO transcription factors on cell biology and believed that the FoxO signaling pathway had a potential role in the treatment and prevention of human cancer targets [17].

After that, we further analyzed the COVID-19-related GO terms with the help of GSEA and obtained that the COVID-19 lung tissue gene set was enriched in the positive regulation of myeloid leukocyte cytokine production involved in immune response, programmed necrotic cell death, translesion synthesis, necroptotic process, and condensed nuclear chromosome. Yang et al. found through research that secondary necrosis occurred after apoptosis, necrosis would actively trigger an immune response, and apoptosis would induce immune tolerance and confirmed that the pathway of programmed cell death could cause systemic lupus erythematosus disease [18]. Patel et al. introduced the role of translesion synthesis in cancer in their research and discussed the identification and development of inhibitors for various translesion synthesis DNA polymerases. Finally, they concluded that translesion synthesis inhibitors could become a new tumor treatment target [19]. Dhuriya and Sharma mentioned that necroptosis was another mode of regulating cell death and required the protein RIPK 3 and its substrate MLKL. And through mouse experiments, they confirmed that RIPK/MLKL-mediated necroptosis had a vital function in the destructive inflammation during viral infection [20].

We constructed a PPI network for upregulation and downregulation of DEGs through STRING and screened out 3 upregulated hub genes (ISG15, FN1, and HLA-G) and 4 downregulated hub genes (FOXP3, CXCR4, MMP9, and CD69). The protein encoded by the gene ISG15 is a ubiquitin-like protein that binds to target proteins in the cell; after that, interferon-*α* and interferon-*β* are activated. Perng and Lenschow discussed the role of ISG15 in regulating host damage and repair responses and immune responses and provided new insights for ISG15 in shaping the host's response to viral infections [21]. Cardoso et al. mentioned that 11 genes including FN1 were related to endometriosis [22]. HLA-G is the HLA class I heavy chain catalog. Curigliano et al. pointed out that this gene was not only involved in expression in the placenta but also melanoma, head and neck, lung, genitourinary system, gastrointestinal tract, and breast cancer [23]. Through the study of the immune tolerance function of this gene in tumors, it is concluded that HLA-G is an effective immunosuppressive molecule, and eliminating the expression of HLA-G in cancer cells is of great significance for anticancer therapeutic effects. FOXP3 is a protein-coding gene, and research has pointed out that this gene is involved in expression in diseases, such as immune regulation disorders [24], polyendocrine diseases [25], bowel diseases [26], and X-linked and fetal edema [27]. CXCR4 is a member of the C-X-C chemokine receptor family and is involved in the regulation of many types of cancer. Jiang et al. analyzed 3637 patients with gastrointestinal cancer by meta-analysis, and overexpression of CXCR4 was closely associated with the overall survival rate of patients with gastrointestinal cancer and could be used as a prognostic indicator for patients with gastrointestinal cancer [28]. Radosinska et al. found that MMP2 and MMP9 were related to heart failure, and the levels of MMP2 and MMP9 could be used as indicators of treatment effect for patients with heart failure [29]. CD69 is a marker of white blood cell activation. Early studies have shown that this gene is involved in expression in lung tissues of patients with asthma and eosinophilic pneumonia. Hasegawa and Nakayama proved through mouse experiments that the arthritis inflammation and airway inflammation in mice lacking the CD69 gene were significantly reduced, suggesting that this gene might become a new target for the treatment of arthritis and asthma [30]. This study still has limitations. First, the expression profile of DEGs needs to be verified by clinical samples. Secondly, further experimental study is required to confirm the function of the hub genes in COVID-19.

GSE151764 was acquired from the GEO database and selected 34 groups of COVID-19 lung tissues and 17 other groups of lung tissues as sample data for analysis. The samples were analyzed by limma package in R, and 84 upregulated DEGs and 46 downregulated DEGs were identified. After that, the GO terms and KEGG pathways enriched by these DEGs were analyzed with the use of the DAVID database, further enrichment analysis of the COVID-19 gene set was performed through GSEA, and the effects of these enriched pathways in other diseases were studied. Finally, based on the online STRING tool, a PPI network for DEGs was constructed, and three upregulated hub genes (ISG15, FN1, and HLA-G) and four downregulated hub genes (FOXP3, CXCR4, MMP9, and CD69) were predicted. Our research provides effective targets for the early diagnosis, treatment, and prevention of COVID-19 treatment.

## Data Availability

The datasets used and/or analyzed during the current study are available from the corresponding author upon reasonable request.

## Supplementary Materials

Supplementary Materials Table S1: list of the top 10 significant DEGs in COVID-19 based on GSE151764.
