# Supplementary material for: Identification of Differentially Expressed Genes in COVID-19 and Integrated Bioinformatics Analysis of Signaling Pathways
Source: Genet Res (Camb). 2021 Dec 24;2021:2728757. doi: 10.1155/2021/2728757 (PMC8710042; doi:10.1155/2021/2728757)
Supplement: Supplementary Materials — Table S1: list of the top 10 significant DEGs in COVID-19 based on GSE151764. [file 2728757.f1.docx]

**Supplementary materials**

**Table S1** List of the top 10 significant DEGs in COVID-19 base on GSE151764

| Up-regulated genesin COVID-19 | Down-regulated genes in COVID-19 |
| --- | --- |
| PPFIBP1 | FOXP3 |
| SFTPA2 | AC011558.5 |
| PKD1P1 | CD226 |
| IFI6 | OR7E43P |
| OAS1 | CD1C |
| COL1A1 | CD69 |
| KIAA0101 | OR7E23P |
| PAPOLA | PGF |
| SFTPA1 | ASRGL1 |
| ONECUT2 | RP11-119H12.1 |

Abbreviations: COVID-19, Corona Virus Disease 2019; DEGs, differentially expressed genes.
